# Hydrochlorate of Cocaine in Catarrhal Ophthalmia

**Published:** 1885-05

**Authors:** R. H. Taylor

**Affiliations:** Griffin, Ga.


### HYDROCHLORATE OF COCAINE IN CATARRHAL OPHTHALMIA.

BY R. H. TAYLOR, M. D., GRIFFIN, GA.

Perhaps no remedy ever introduced to the profession has been so enthusiastically advocated, so extensively studied, and so much discussed in such a short time, as hydrochlorate of cocaine.

I will, therefore, be as concise as possible, and not bore your readers with a reiteration of what has already been said, and so well said, too; but I wish, briefly, to call attention to its use in the treatment of acute catarrhal ophthalmia. Noticing the records of cases where it has been used as a local anæsthetic, I observed that it exerted a powerful, and almost immediate, influence on the blood-vessels of the conjunctiva, reducing their caliber and diminishing the blood supply. This led me to give it a trial in acute inflammatory conditions of the membrane. I have now used it in several cases of acute catarrhal conjunctivitis, and find its effects far beyond my most sanguine expectations.

The first application relieved the pain in about five minutes, and it was applied every five or ten minutes until four or five applications were made. Thus used on my first case, immediately before bed time, and within the *first twelve* hours of the attack, was sufficient.

Nothing else was ever required, and the next day the patient reported himself well.

The next case was not seen so promptly in the beginning of the attack, and it required several days to accomplish a cure, but the relief of pain, photophobia and profuse lachrymation was as prompt as in the first case.

After the eye is brought under its influence, three or four applications daily has, in my hands, been sufficiently often to keep up the anæmic condition of the mucous membrane, and the disease is promptly starved out.

My next case came to consult me for a foreign body in the eye, which, he said, had been in his eye for two or three weeks. I examined the eye and found a slight scratch on the cornea, which was about well. On inverting the upper lid, I found the lower edge of the cartilage thickly studded with very fine granules, and quite red. I applied the hydrochlorate of cocaine, as in the other cases, three or four times daily, only in this case I applied it with a camel's hair pencil instead of dropping into the eye.

In about two weeks I had the satisfaction of seeing all the granules disappear. I have not used it very extensively, 'tis true, but those few cases where I have used it are so encouraging that I would like to have others try it thoroughly. If it is not absolutely curative in every case, it will at least relieve one of the most distressing symptoms, and give the patient comfort, while astringents may be employed at the same time.

Surely I would not make positive claims on so few cases, but I think it deserves thorough trial.

